# Post-Lumbar Puncture Headache—Does Hydration before Puncture Prevent Headache and Affect Cerebral Blood Flow?

**DOI:** 10.3390/jcm8101710

**Published:** 2019-10-17

**Authors:** Magdalena Nowaczewska, Beata Kukulska-Pawluczuk, Henryk Kaźmierczak, Katarzyna Pawlak-Osińska

**Affiliations:** 1 Department of Pathophysiology of Hearing and Balance, Ludwig Rydygier Collegium Medicum in Bydgoszcz Nicolaus Copernicus University, M. Curie 9, 85-090 Bydgoszcz, Poland; osinskak1@wp.pl; 2 Department of Neurology, Ludwig Rydygier Collegium Medicum in Bydgoszcz Nicolaus Copernicus University, M. Curie 9, 85-090 Bydgoszcz, Poland; kikneurol@cm.umk.pl; 3 Department of Otolaryngology, Head and Neck Surgery, and Laryngological Oncology, Ludwik Rydygier Collegium Medicum in Bydgoszcz Nicolaus Copernicus University, M. Curie 9, 85-090 Bydgoszcz, Poland; orlamb@cm.umk.pl

**Keywords:** cerebral hemodynamics, cerebral blood flow, transcranial Doppler, neurosonology, post-lumbar puncture headache, flow velocity, intracranial pressure, pulsatility index, hydration, fluid, intracranial hypotension

## Abstract

Headache is a common complication after diagnostic lumbar puncture (DLP). We aimed to check whether hydration before puncture influences the incidence of post-lumbar puncture headache (PLPH) and affects cerebral blood flow. Ninety-nine patients enrolled for puncture were assigned to a group with (*n* = 40) or without hydration (*n* = 59). In the hydration group, 1000 mL 0.9% NaCl was infused and a minimum of 1500 mL oral fluids was recommended within the 24 h before puncture. A Transcranial Doppler (TCD) was performed before and after DLP. Mean velocity (Vm) and pulsatility index (PI) were measured in the middle cerebral arteries (MCAs). PLPH occurred in 28 patients (28.2%): six (15.4%) from the hydrated and 22 (37.3%) from the non-hydrated group (*p* < 0.023). Patients with PLPH were younger (*p* < 0.014) and with headaches in their histories (*p* < 0.036) compared with the non-headache group. Vm values in both MCAs after puncture were significantly lower than before puncture in all patients. In the PLPH group, Vm in MCAs before puncture were significantly higher and the PI was lower than in the non-headache group. Our findings suggest that hydration of patients within 24 h before puncture prevented PLPH. Twenty-four hours after puncture, significant decreases in Vm were observed in the MCAs of all patients. Low baseline values of PI and high Vm predisposed patients to PLPH.

## 1. Introduction

Diagnostic lumbar puncture (DLP) is a frequently performed medical procedure, which is important in the diagnosis of nervous system diseases. Headache is the most common complication after DLP, with frequency rates ranging from 3% to 40% of patients [1,2,3,4]. Post-lumbar puncture headache (PLPH) probably results from the leakage of cerebrospinal fluid into the epidural cavity through the post-puncture opening in the dura mater. The loss of cerebrospinal fluid causes a drop in intracranial pressure (ICP), leading to the compensative dilatation of cerebral vessels and hence, the headache [1,2,3,5]. Transcranial Doppler ultrasonography (TCD) makes it possible to assess blood flow in the intracranial arteries non-invasively. TCD parameters are influenced both by changes in cerebral vessel diameters and by fluctuations in ICP [6,7,8]. DLP results in significant changes in cerebral blood flow (CBF) that can be visualized by TCD [9,10,11]. The role of fluid supplementation in the prevention of PLPH remains unclear [12]. The results of a few current studies have unambiguously confirmed that hydration of patients after puncture prevents PLPH [13,14,15]. This is why we decided to check whether hydration before puncture influences the incidence of PLPH and causes changes in CBF which are reflected in TCD.

## 2. Experimental Section

The prospective parallel study involved patients hospitalized in the Neurology Clinic who required scheduled DLP. The exclusion criteria included emergency lumbar punctures, inadequate temporal windows, stenosis of the middle cerebral arteries, hemodynamically significant stenosis of the internal carotid arteries, atrial fibrillation, general severe medical conditions, heart, kidney, and respiratory failures, unconsciousness, speech disorders, and patient immobilization. Patients enrolled for puncture were randomly assigned following simple randomization procedures (using the basic random number generator in Excel) to one of two groups: one containing patients with hydration and one without hydration. In the hydration group, intravenous 1000 mL 0.9% NaCl was infused within the 24 h before puncture and an increased amount of oral fluids was also recommended (minimum 1500 ml). A TCD was performed just before DLP and 24 h after DLP. The lumbar puncture was performed according to the standard procedure in a supine position, by one of the 11 doctors working in the clinic. On the day of puncture, plasma osmolality was determined in the morning. PLPH was diagnosed according to the International Classification of Headaches (ICH-III beta) [16]. The presence and severity of PLPH was assessed using a numerical scale (numeric rating scale, NRS) every day for five days. All the procedures were approved by the Local Ethics Committee of the Ludwik Rydygier Collegium Medicum in Bydgoszcz (KB/285/2015). The subjects gave their informed consent before the start of any procedure.

Lumbar puncture was performed in the lateral recumbent position in the space between L4 and L5, or between L5 and S1, using a 22-gauge needle (BD Spinal Needle Quincke Type Point). The volume of the cerebral fluid collected ranged from 3 mL to 6 mL. Punctures were performed between 11 a.m. and midday.

TCD examinations were performed with a Nicolet Sonara transcranial doppler system (producer Viasys Healthcare) and a 2 MHz probe. Patients were examined in the supine position. After finding the temporal window, the middle cerebral artery was identified. The probe was positioned in such a way as to obtain the highest possible velocity of the tested vessel. Mean velocity (Vm) and Gosling’s pulsatility index (PI) were measured at a 54–56 mm depth in the right and left middle cerebral artery (MCA). TCD tests were performed by a physician experienced in the field of neurosonology who was not informed about the presence and severity of PLPH or the hydration of the patient. 

The results are presented as mean ± standard deviation (SD). Mean values close to normal distribution were compared for independent samples with Student’s *t* test. When the distribution was significantly different from the normal distribution, differences between the groups were checked using the non-parametric Mann–Whitney U test. Dependent variables were checked using the Student’s *t* test for dependent samples. Proportions in the groups were evaluated using the χ^2^ test. Correlation between the two groups was calculated with Spearman’s rank correlation coefficient. The Shapiro–Wilk test was used to check for normality. Continuous variables were compared between the four groups with an analysis of variance (ANOVA) or the Kruskal-Wallis test, as appropriate. The Dunn test was used for post-hoc comparisons, with *p*-values adjusted using Holm’s method. Categorical variables were compared with Fisher’s exact test. When categorical variables significantly differed between the four groups, pairwise Fisher’s exact tests were used for post-hoc comparisons, with *p*-values adjusted using the Holm’s method. Statistical significance was set at *p* < 0.05, two-tailed. All the calculations were made in R software. 

## 3. Results

Ninety-nine patients were enrolled for this study, including 45 men and 54 women. The mean age was 42.5 ± 14.48 years (range: 18 to 79 years). Patients were diagnosed due to the following: demyelinating disease (21 patients), headaches (14), mononeuropathy (11), abnormal outbreaks in head MR (11), transient ischemic attack (11), polyneuropathy (5), vertigo (5), loss of consciousness (7), and other reasons (epilepsy, transient global amnesia, tremor, syphilis).

Forty patients were hydrated 24 h before puncture, and in 59 patients punctures were performed without prior hydration. Post-puncture headache occurred in 28 patients (28.2%): six (15.4%) in the hydrated group and 22 (37.3%) in the non-hydrated group (*p* < 0.023). Patients with PLPH were significantly younger and more frequently reported headaches in their medical histories compared with the group without headache. The characteristics of the patients in each group are presented in Table 1 and Table 2.

There were no significant differences in Vm between the right and left MCAs, before and after puncture, while PI values were significantly different between the cerebral hemispheres. PI was higher on the right side before and after the puncture (*p* < 0.001).

Vm values in both MCAs after puncture were significantly lower than before puncture in all the groups of patients, while PI after puncture increased only in the group of patients with post-puncture headache (Table 2). Percentage changes in Vm (dVm) and PI (dPI) after puncture were highest in the group of patients with PLPH who had not been hydrated (Table 2).

In patients with PLPH, the values of Vm in both MCAs before puncture were significantly higher and PI values were lower than in the group without headache, while no significant differences in Vm and PI values after puncture were observed between the groups (Table 2). The highest Vm values before puncture were found in the hydrated PLPH patients. The Vm values after puncture were higher in the hydrated group than in the non-hydrated group; there were no significant differences in PI between the hydrated and non-hydrated group, before and after the puncture (Table 2).

Moreover, there were no significant differences in the osmolality of the serum before puncture between the hydrated, non-hydrated, PLPH, and non-PLPH group; however, the average osmolality in all the groups was higher than the laboratory standard (Table 2).

The logistic regression model for PLPH (PLPH occurrence as a dependent variable) showed more frequent occurrence of PLPH in patients who were younger (*p* < 0.014), not hydrated (*p* < 0.007) and had a history of headaches (*p* < 0.036).

## 4. Discussion

**Figure 1:**
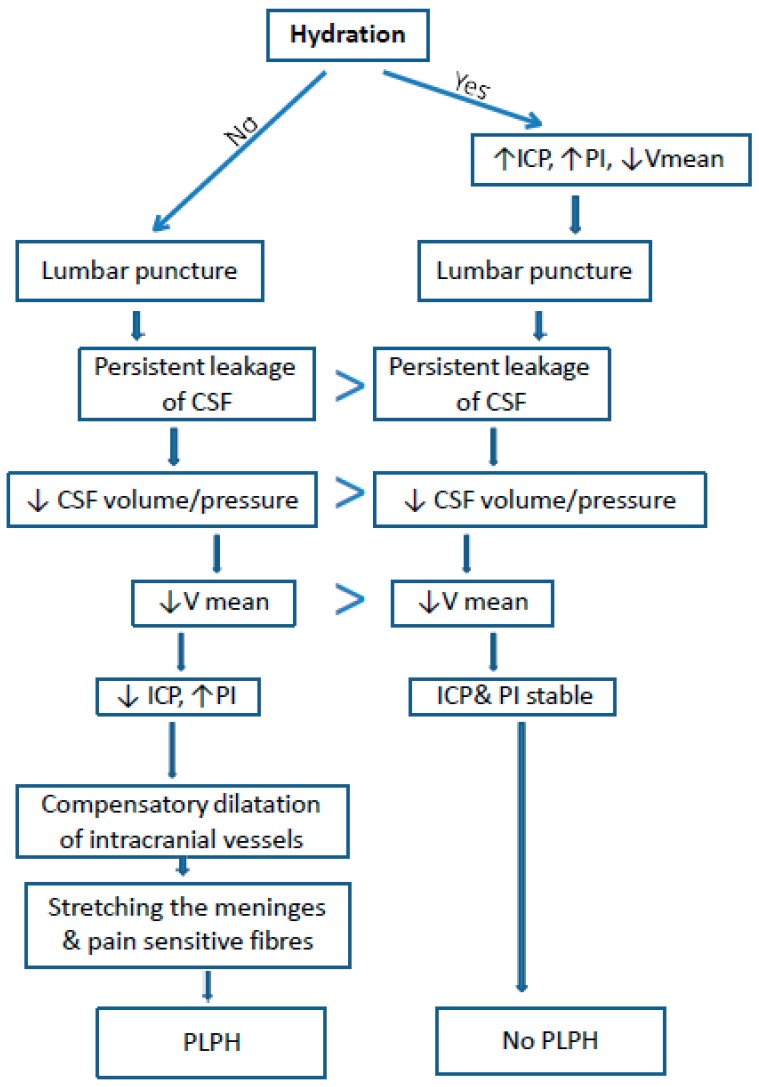
Proposed mechanism of PLPH pathogenesis and the influence of hydration on the incidence of PLPH and cerebral blood flow (based on our study). ICP–intracranial pressure, PI–Gosling’s pulsatility index, V mean–mean velocity, CSF–cerebro-spinal fluid, PLPH–post lumbar puncture headache.

Post-lumbar puncture headache is probably caused by leakage of the cerebrospinal fluid through the post-puncture opening, which leads to a decrease in intracranial pressure and dilation of the cerebral vessels [1,2,3,5]. The change of the above parameters may affect the cerebral blood flow assessed by TCD: the dilatation of the cerebral arteries causes a decrease in the flow velocity [6,7,8] (Figure 1). The pulsatility index is an indirect indicator of the ICP changes. The value of the PI illustrates the resistance of a vascular bed (supplied by a given artery) when examined by TCD. There is a linear relationship between changes in PI and ICP. A decrease in PI may result both from lowered ICP and from vascular dilatation [7,8].

In our previous study, we showed that patients with a lower baseline PI and higher Vm developed PLPH more often [9]. This was recently confirmed by other authors [10]. From previous studies we know that the PI positively correlates with ICP—a PI change of 2.4% is reflected by a 1 mmHg change in ICP [17]. Therefore, it can be assumed that lower PIs correspond to a lower ICP. This means that patients with lower baseline ICP develop PLPH more frequently. One of the factors that may affect ICP is hydration; therefore, we assumed that mild dehydration of patients before lumbar puncture may influence the incidence of PLPH. Thus, we decided to investigate how the hydration of patients before the puncture affects PI, Vm, and the occurrence of PLPH.

The results of several current studies describe no significant effect of patient hydration on the occurrence of post-lumbar puncture headache [12,13,15]. However, most patients were hydrated after puncture or directly before the procedure. Dietrich hydrated patients for five days after DLP and did not notice a difference in the occurrence of PLPH [13]. The patients involved in the study by Gupta et al. were hydrated directly before and after puncture. They were given 500 mL of saline intravenously 30 min before and after puncture and showed no significant reduction in PLPH compared to the non-hydrated group. Nevertheless, they noticed a significant reduction in pain severity [15]. The effects of intravenous and oral hydration within the 24 h before puncture on the occurrence of PLPH have not been described by any author. 

In our study, we showed that in the group of patients hydrated before puncture, PLPH was significantly less frequent than in the group without hydration. In further analyses of the effect of hydration on CBF and PLPH, we additionally used TCD parameters. 

In all the patients, puncture was followed by a decrease in Vm in both MCAs, while PI after puncture increased only in the PLPH group. Most likely, the ICP decreased after the puncture which led to the dilatation of the arteries and the decrease in Vm. This decrease was the highest in the group with PLPH. V correlates linearly with PI and ICP (when PI and ICP decreases, there is a Vm increase), a large decrease in Vm in the PLPH group probably caused compensatory vasoconstriction in the microcirculation, which, in turn, caused PI growth. This confirms the theory of PLPH pathophysiology according to which, in patients with PLPH, additional cerebrospinal fluid leakage through the post-puncture opening causes a greater decrease in ICP, and thus, a greater decrease in Vm in the MCA. 

In the group of patients with PLPH, baseline Vm values (before puncture) were higher and PI values were lower than in the group without PLPH. It is known that with the increase of ICP, PI increases and Vm decreases. Therefore, it can be concluded that in patients who developed PLPH, the baseline ICP was lower than in the group without PLPH. This could be associated with a greater dehydration before puncture in this group. The baseline low ICP led to PLPH development after puncture, while in the group with higher baseline ICP, despite the reduction of V after puncture, headache did not occur. It is possible that a higher pre-puncture ICP protected patients from excessive vasodilatation and decreased ICP (Figure 1). Nevertheless, that lower PIs and higher Vm in the group of PLPH patients may have resulted from young age can be excluded because Vm decreases with age, while PI increases [18].

It is worth emphasizing that in the group of patients who were hydrated before puncture, post-puncture Vm values were higher than in the group without hydration. This is probably why these patients less frequently noticed the decrease of ICP values and PLPH development. 

Can the occurrence of PLPH be predicted based on TCD parameters before the puncture? Movafy showed that pre-puncture Vm was the parameter with the greatest accuracy for predicting PLPH, with a cutoff of Vm > 68.4 cm/s. Our study confirms these observations. In addition, similarly to other authors, we confirmed the correlation between PLPH with age and a history of headaches [1,2].

In our patients, an interhemispheric difference in PI was found before and after puncture, while mean Vm was not significantly different between the right and left MCA. Gobel et al. noticed that patients who develop post-lumbar puncture headache were characterized before lumbar puncture by a significant lateralization of Vm of the MCAs—the flow velocity in the right artery predominated significantly. Unfortunately, they did not evaluate PI value, only Vm. It is difficult to explain our results clearly; this requires further observation and more accurate research.

PLPH is only one type of headache; however, PLPH patients often have a medical history of various types of headaches. The level of hydration affects headaches in general. It is known that dehydration promotes the occurrence of migraine and other types of headaches [19,20]. Spigt noticed that increased hydration reduced the number of pain days in patients with migraine and tension headache [19]. Perhaps pre-puncture hydration prevents the occurrence of PLPH not only by a mechanical CBV increase but also by means of another mechanism. 

Although, in our study, we showed that pre-puncture hydration reduced the incidence of PLPH, we did not find differences in plasma osmolality between groups. Perhaps small and rapid changes in the hydration in our patients were not detectable by changes in plasma osmolality. This was also confirmed by other authors [21]. Interestingly, in all the groups, plasma osmolality values were higher than the laboratory norm, which may indicate a dehydration problem in all the patients before DLP. Dehydration may be related with hospitalization, stress, or fasting before the planned examinations (e.g., MRI).

The limitation of this study is the fact that TCD measures only the flow of velocity and not the absolute CBF value. The correlation between CBF and flow velocity is variable. Cerebral blood velocity is an adequate surrogate of absolute flow only if the insonated vessel maintains constant vessel diameter in the experimental conditions. Blood flow velocity is further influenced by several factors, including arterial blood pressure, ICP, hematocrit, PaCO2 and the status of autoregulation, thus making a direct comparison of flow velocity and CBF difficult. Moreover, it is worth noting that a correlation between PI and ICP has been found in the context of intracranial hypertension (after trauma, intracranial heamorrhage, cerebral mass lesion and hydrocephalus) and is strongest when ICP is over 20 mm [7,8,17]. There is no study of PI in patients with intracranial hypotention or dehydration.

Another limitation of the study may be the fact that the hydration level of the patients was evaluated only by a single measurement of plasma osmolality before DLP, while urine osmolality and gravity was not assessed, nor did we assess any hydration level parameters after puncture.

## 5. Conclusions

Our findings suggest that oral and intravenous hydration of patients within the 24 h before puncture prevented PLPH. The changes of TCD parameters partially confirm the theory of PLPH pathogenesis as a consequence of cerebrospinal fluid leakage through the post-puncture opening, with subsequent vasodilatation and ICP decrease. A history of headaches, low baseline values of PI, and high Vm predispose patients to PLPH occurrence.

## Figures and Tables

**Table 1:** Clinical characteristics of hydrated and non-hydrated patients; patients who developed post-lumbar puncture headache (PLPH) and PLPH-free individuals.

Parameter	Hydrated Group	Non-Hydrated Group	*p*-Value	PLPH	PLPH-Free	*p*-Value
	*n* = 40	*n* = 59		*n* = 28	*n* = 71	
Age (mean ± SD)	41.5 ± 13.7	43.3 ± 15.1	0.538	37.2 ± 11.85	44.86 ± 14.94	0.009
Sex: Men, *n* (%)	19 (47.5)	26 (44.1)	0.838	9 (32.1)	36 (51.4)	0.116
History of headache, *n* (%)	16 (40.0)	20 (33.9)	0.671	15 (53.6)	20 (28.6)	0.034
Plasma osmolality	299.80	298.98	0.817	299.64	299.39	0.978
Hypertension *n* (%)	12 (30.0)	9 (15.3)	0.087	4 (14.3)	17 (24.3)	0.414
Hipercholesterolemia	9 (22.5)	12 (20.3)	0.807	4 (14.3)	16 (22.9)	0.416
Smoking *n* (%)	14 (35.0)	15 (25.4)	0.370	6 (21.4)	22 (31.4)	0.458
Mood disorders *n* (%)	5 (12.5)	9 (15.3)	0.776	5 (17.9)	8 (11.4)	0.510
PLPH n (%)	6 (15.4)	22 (37.3)	**0.023**	-	-	
NRS	4.67	5.00	0.699	-	-	

NRS-numeric rating scale.

**Table 2:** Characteristics and transcranial Doppler (TCD) parameters before and after puncture in groups of patients who developed post-lumbar puncture headache (PLPH) and in PLPH-free individuals, depending on the hydration.

	PLPH		PLPH-Free		*p*-Value
	Hydrated Group	Non-Hydrated Group	Hydrated Group	Non-Hydrated Group	
	*n* = 6	*n* = 22	*n* = 33	*n* = 37	
Age mean (SD)	35.00 (8.10)	37.77 (12.78)	43.00 (14.26)	46.51 (15.52)	0.076
Plasma osmolality mean (SD)	297.00 (12.54)	300.36 (12.81)	300.76 (12.27)	298.14 (12.47)	0.775
Headache in medical history, *n* (%)	6 (100.0)	9 (40.9)	9 (27.3)	11 (29.7)	0.006
Pre-puncture Vm, MCA R, mean (SD)	84.02 (23.84)	74.51 (11.82)	66.51 (14.12)	65.14 (17.36)	0.013
Pre-puncture Vm, MCA L, mean (SD)	81.90 (23.05)	74.20 (13.85)	67.31 (14.37)	64.00 (15.88)	0.016
Pre-puncture PI, MCA R, median	0.78 (0.74, 0.86)	0.81 (0.75, 0.89)	0.92 (0.81, 0.97)	0.91 (0.83, 1.02)	0.017
Pre-puncture PI, MCA L, median	0.78 (0.70, 0.85)	0.80 (0.70, 0.86)	0.87 (0.79, 0.99)	0.87 (0.79, 0.98)	0.02
Post-puncture Vm, MCA R, mean (SD)	72.43 (22.71)	55.80 (11.62)	63.60 (14.31)	60.89 (15.71)	0.074
Post-puncture Vm, MCA L, mean (SD)	75.30 (17.16)	54.32 (10.99)	64.06 (11.99)	62.07 (14.31)	0.003
Post-puncture PI, MCA R, median	0.70 (0.65, 0.74)	0.90 (0.84, 0.97)	0.93 (0.82, 1.04)	0.89 (0.78, 1.08)	0.121
Post-puncture PI, MCA L, median	0.73 (0.68, 0.79)	0.85 (0.75, 0.95)	0.91 (0.78, 0.98)	0.88 (0.78, 0.97)	0.301
dVm R median	−14.02 (−22.94, −0.71)	−25.02 (−32.50, −15.92)	−6.85 (−13.64, 4.49)	−8.49 (−12.30, 2.12)	<0.001
dVm L mean (SD)	−5.85 (12.90)	−25.41 (15.06)	−3.68 (12.02)	−1.80 (12.79)	<0.001
dPI R mean (SD)	−7.50 (9.63)	10.50 (10.89)	1.16 (10.08)	−0.00 (13.33)	0.001
dPI L mean (SD)	−3.31 (14.23)	11.40 (16.41)	0.93 (14.31)	−1.96 (14.36)	0.008
Sex: Men *n* (%)	1 (16.7)	8 (36.4)	18 (54.5)	18 (48.6)	0.285
NRS, mean (SD)	4.67 (2.58)	5.00 (1.63)	NA	NA	0.699
Pre-puncture Vm, MCA R (cm/s)	76.55 ± 15.16		65.79 ± 15.81		0.003
Post-puncture Vm, MCA R (cm/s)	59.36 ± 15.78		62.17 ± 15.02		0.425
*p*-Value	0.000		0.001		
Pre-puncture Vm, MCA L (cm/s)	75.85 ± 16.06		65.56 ± 15.17		0.006
Post-puncture Vm, MCA L (cm/s)	58.81 ± 15.01		63.01 ± 13.21		0.203
*p*-Value	0.000		0.013		
Pre-puncture PI, MCA R	0.82 ± 0.13		0.93 ± 0.16		0.002
Post-puncture PI, MCA R	0.87 ± 0.16		0.93 ± 0.17		0.149
*p*-Value	0.014		0.836		
Pre-puncture PI, MCA L	0.79 ± 0.14		0.90 ± 0.16		0.002
Post-puncture PI, MCA L	0.85 ± 0.18		0.88 ± 0.16		0.411
*p*-Value	0.021		0.402		

MCA–middle cerebral artery, PI–Gosling’s pulsatility index, Vm–mean velocity, dVm–percentage changes in Vm.
